# Non-invasive predictors of prognosis of Asian patients with histopathologically-confirmed lean nonalcoholic fatty liver disease

**DOI:** 10.1186/s12876-020-01509-3

**Published:** 2020-11-04

**Authors:** Soichi Iritani, Norio Akuta, Yusuke Kawamura, Akira Kajiwara, Kayoko Kasuya, Shunichiro Fujiyama, Hitomi Sezaki, Tetsuya Hosaka, Masahiro Kobayashi, Mariko Kobayashi, Satoshi Saito, Fumitaka Suzuki, Yasuji Arase, Kenji Ikeda, Yoshiyuki Suzuki, Hiromitsu Kumada

**Affiliations:** 1 Department of Hepatology, Toranomon Hospital and Okinaka Memorial Institute for Medical Research, 2-2-2 Toranomon, Minato-ku, Tokyo, 105-8470 Japan; 2 Liver Research Laboratory, Toranomon Hospital, Tokyo, Japan

**Keywords:** NAFLD, NASH, Lean NAFLD, Overall survival, Prognosis, Mortality, Fibrosis, NAFLD fibrosis score, NFS, Liver cancer

## Abstract

**Background:**

The prognostic factors of morbidity and mortality in patients with lean NAFLD (body mass index < 25.0 kg/m^2^) are unknown.

**Methods:**

In this retrospective study, 446 Japanese patients with histopathologically-confirmed NAFLD (lean NAFLD, n = 170) were followed for liver events, cardiovascular events, type 2 diabetes mellitus, and non-liver malignancies. The median observation period was 4.6 years. We also investigated the predictors of severe fibrosis (stage 3–4) and mortality in lean NAFLD patients.

**Results:**

Glycolipid metabolic markers, liver function tests, NAFLD fibrosis score (NFS), and histological scoring were significantly lower in lean NAFLD patients than in non-lean NAFLD. The incidence of liver cancer was higher while that of T2DM was lower in lean NAFLD. Kaplan–Meier analysis showed no significant difference in overall survival between the lean and non-lean NAFLD. Multivariate analysis of data of lean NAFLD identified NFS ≥ − 1.455 as significant independent predictor of severe fibrosis, while history of liver cancer and NFS ≥ − 1.455 were predictors of overall survival.

**Conclusions:**

Although patients with lean NAFLD have better histopathological and biochemical profile compared to patients with non-lean NAFLD, the prognosis is not different between the two groups. Lean NAFLD patients with NFS ≥ − 1.455 or history of liver cancer should be monitored carefully during follow-up.

## Background

Nonalcoholic fatty liver disease (NAFLD) is the most common liver disease worldwide [1–6]. Liver pathology ranges from the typically benign nonalcoholic fatty liver to nonalcoholic steatohepatitis (NASH), but it sometimes progresses to liver cirrhosis, hepatocellular carcinoma, and liver failure [7]. In Japan, follow-up of obese patients with NAFLD is mandatory, with the aim of reducing the chance of insulin resistance and preventing disease progression [8]. Most lean persons with NAFLD display insulin resistance and altered body fat distribution even though they have less severe metabolic disturbances than overweight NAFLD. Lean NAFLD has been defined as body mass index (BMI) < 25.0 kg/m^2^ [9]. In Japan, we often encounter lean NAFLD patients in daily practice.

The prognosis of lean NAFLD is considered to be better than that of obese NAFLD [10]. However, a higher overall mortality was reported in patients with lean NAFLD compared with obese NAFLD patients in an 11-year follow-up study, despite presentation with a healthier metabolic profile, including low insulin resistance [11]. Moreover, lean NAFLD has been reported to be independently associated with increased risk of all-cause and cardiovascular mortality compared with lean individuals without NAFLD [12]. To date, the prognosis of lean NAFLD is still unknown in Japan.

The present study was designed to define the clinical features of histopathologically-confirmed lean NAFLD patients. Furthermore, by using non-invasive tests, we analyzed the data to obtain clinically meaningful prognostic factors and predictors of severe fibrosis and survival.

## Methods

### Patients

Patients admitted to our hospital with liver dysfunction and/or fatty liver, diagnosed by clinical examination, laboratory tests and abdominal ultrasonography, between 1976 and 2019, underwent liver biopsy. Histopathological examination confirmed the diagnosis of NAFLD in 446 of these patients. These patients included those in whom histopathological examination showed microscopic changes consistent with steatosis in at least 5% of hepatocytes and patients with history of alcohol intake of < 20 g/day. The median duration of follow-up from diagnosis to death or last visit was 4.6 years (range 0.0–43.5 years). The clinical features of the patients at the time of histopathologic diagnosis of NAFLD are summarized in Table 1. We excluded patients with (1) underlying liver disease (e.g., viral hepatitis, autoimmune hepatitis, drug-induced liver disease, or primary biliary cholangitis); (2) systemic autoimmune diseases (e.g., systemic lupus erythematosus or rheumatoid arthritis); and (3) metabolic diseases (e.g., hemochromatosis, α-1-antitrypsin deficiency, or Wilson’s disease).

**Table 1:** Background factors at the time of liver biopsy

n	446
Age	52 (18–87)
Sex, male/female	268/178
Body mass index (kg/m^2^)	26.3 (18.1–42.4)
History of liver cancer, yes/no	29/416
History of non-liver malignancy, yes/no	39/405
Type 2 diabetes mellitus, yes/no	147/297
Dyslipidemia, yes/no	151/294
Hypertension, yes/no	200/246
Hyperuricemia, yes/no	46/399
Smoking, yes/no	98/338
Albumin (g/dL)	4.1 (2.8–6.9)
Aspartate aminotransferase (IU/L)	44 (12–378)
Alanine aminotransferase (IU/L)	69 (13–783)
Gamma-glutamyl transpeptidase (IU/L)	71 (11–990)
Hemoglobin (g/dL)	14.6 (6.5–18.7)
Platelet count (× 104/μL)	21.2 (4.0–47.1)
Triglyceride (mg/dL)	139 (31–1088)
Total cholesterol (mg/dL)	203 (101–370)
High-density lipoprotein cholesterol (mg/dL)	45 (14–86)
Low-density lipoprotein cholesterol (mg/dL)	121 (27–243)
Fasting blood sugar (mg/dL)	103 (65–287)
Glycated hemoglobin (%)	6.0 (4.3–12.6)
Ferritin (ng/mL)	228 (1–2067)
NAFLD fibrosis score	− 1.844 (− 7.060 to 3.394)
Genetic variation (n = 314)	
*PNPLA3* rs738409, CC/CG/GG/not done	54/129/131/132
*TM6SF2* rs58542926, CC/CT/TT/not done	237/70/7/132
*HSD17B13* rs6834314, AA/AG/GG/not done	161/132/25/128
Histopathological findings (n = 446)	
Steatosis, 5%–33%/ 33%–66%/> 66%	164/167/112
Ballooning, none/few cells/many cells	40/283/120
Lobular inflammation, no foci/< 2 foci/2–4 foci/> 4 foci per 200 × field	28/254/147/14
NAFLD activity score, ≤ 2/3,4/≥ 5	35/190/218
Fibrosis stage, 0/1/2/3/4	51/182/69/110/34

Data are number of patients or median (range) values

The study was conducted in compliance with the International Conference on Harmonization Guideline for Good Clinical Practice (E6) and the 2013 Declaration of Helsinki. The study protocol was approved by the Toranomon Hospital Institutional Review Board (#953). Written informed consent for liver biopsy was provided by all patients.

### Diagnosis and follow-up

In this study, we selected the following liver-related events for study outcome: liver cancer, hepatic encephalopathy, esophagogastric varices with bleeding, ascites, and jaundice. Other outcomes included cardiovascular events (e.g., coronary artery disease, heart valve disease, arrhythmia, heart failure, hypertension, orthostatic hypotension, shock, endocarditis, diseases of the aorta and its branches, disorders of the peripheral vascular system, and stroke), type 2 diabetes mellitus (defined as high fasting blood glucose level ≥ 126 mg/dL, high hemoglobin A1c ≥ 6.5%, use of glucose-lowering agents, or self-reported history of clinical diagnosis), and non-liver malignancy. Mortality was evaluated for all patients. Hematologic and biochemical data were collected at least twice yearly after the diagnosis of NAFLD. Ultrasonography, computed tomography, and/or magnetic resonance imaging studies were conducted at least once annually during the follow-up.

### Liver histopathology

Liver biopsy specimens were obtained using a 14-gauge modified Vim Silverman needle (Tohoku University style; Kakinuma Factory, Tokyo, Japan), a 16-gauge core tissue biopsy needle (Bard Peripheral Vascular, Inc., Tempe, AZ), or surgical resection. Liver biopsy samples > 1.5 cm and/or containing more than 11 portal tracts were considered adequate for examination and diagnosis. The specimen was fixed in 10% formalin and cut into sections, which were subsequently stained with hematoxylin and eosin, Masson trichrome, silver impregnation, or periodic acid–Schiff after diastase digestion. Four pathologists (Dr. Keiichi Kinowaki, Dr. Fukuo Kondo, Dr. Toshio Fukusato, and Dr. Takeshi Fujii), who were blinded to the clinical findings evaluated each of the specimens, and the final assessment was reached by consensus. Steatosis grades 0, 1, 2, and 3 corresponded to steatosis of < 5%, ≥ 5–< 33%, ≥ 33–< 66%, and ≥ 66% of hepatocytes, respectively. Lobular inflammation with no foci, < 2 foci, 2–4 foci, and ≥ 4 foci per 200 × field was scored 0, 1, 2, and 3, respectively. Hepatocyte ballooning of none, few, and many cells was scored as 0, 1, and 2, respectively. NAFLD activity score represents the sum of scores of steatosis, lobular inflammation, and hepatocyte ballooning (range 0–8 points) [13]. Fibrosis stage was defined as 0, 1, 2, 3, and 4 using the defined criteria [13, 14]. NASH was defined according to the fatty liver inhibition of progression (FLIP) algorithm [15].

### Clinical parameters

We included in the analysis various clinicopathologic and genetic parameters that could affect NAFLD prognosis. The NAFLD fibrosis score (NFS), calculated as (− 1.675 + 0.037 × age [years] + 0.094 × BMI [kg/m^2^] + 1.13 × impaired fasting glycemia/diabetes [yes = 1, no = 0] + 0.99 × aspartate aminotransferase/alanine aminotransferase ratio − 0.013 × platelet [× 10^9^/l] − 0.66 × albumin [g/dl]), has been used as a parameter for progression of fibrosis [16]. By applying the low cutoff point (score less than − 1.455), 77% of the patients without significant fibrosis were correctly identified, whereas 22% of patients with a low cutoff point were incorrectly staged [16]. We used the Europeans definition of obesity (BMI ≥ 30 kg/m^2^) [17], while lean NAFLD was defined as BMI < 25.0 kg/m^2^ [9]. Patatin-like phospholipase domain containing protein 3 (*PNPLA3*) rs738409, transmembrane 6 superfamily member 2 (*TM6SF2*) rs58542926, and hydroxysteroid 17-beta dehydrogenase 13 (*HSD17B13*) rs6834314 were genotyped by the TaqMan single-nucleotide polymorphism (SNP) genotyping assay (Applied Biosystems, Foster City, CA).

### Statistical analysis

The baseline characteristics were compared using the Mann–Whitney U test for continuous variables or Fisher’s exact test for categorical variables. The incidence of each event was analyzed during the period from the time of histopathological diagnosis of NAFLD until the last visit or occurrence of event. Overall survival was estimated using the Kaplan–Meier method, and differences between curves were evaluated using the log-rank test. All parameters that showed strong correlation with others were considered confounding factors and excluded from the statistical analysis. The remaining parameters were entered into multivariate analysis using the logistic regression analysis and the Cox proportional hazards model. Statistical significance was set at *P* < 0.05. All statistical analyses were carried out using the EZR software [18].

## Results

### Comparison of background factors stratified by BMI

The baseline characteristics and laboratory data stratified by BMI of the 446 patients are shown in Table 2. The prevalence of type 2 diabetes mellitus, hypertension and hyperuricemia were significantly lower in the lean NAFLD group (BMI < 25). Aspartate aminotransferase, alanine aminotransferase, triglyceride, fasting blood sugar, glycated hemoglobin, ferritin, and NAFLD fibrosis score (NFS) were significantly lower in the lean NAFLD group. Various histopathological findings (e.g., steatosis 3, lobular inflammation 2–3, NAFLD activity score 5–8, and fibrosis stage 3–4) were significantly lower in the lean NAFLD group.

**Table 2:** Background factors stratified by BMI at the time of liver biopsy

	Lean NAFLD^a^	Non-lean NAFLD^b^	*P* value
n	170	276	
Age	53 (18–85)	52 (18–87)	0.549
Sex, male (%)	61.2	59.4	0.765
Body mass index (kg/m2)	23.1 (18.1–24.9)	28.7 (25.0–42.4)	< 0.01
History of liver cancer (%)	8.9	5.1	0.119
History on non-liver malignancy (%)	9.5	8.3	0.730
Type 2 diabetes mellitus (%)	23.5	38.4	< 0.01
Dyslipidemia (%)	28.2	37.5	0.051
Hypertension (%)	31.8	52.9	< 0.01
Hyperuricemia (%)	2.9	14.9	< 0.01
Smoking (%)	22.1	22.7	0.906
Albumin (g/dL)	4.1 (2.8–5.4)	4.1 (3.0–6.9)	0.994
Aspartate aminotransferase (IU/L)	36 (12–312)	51 (15–378)	< 0.01
Alanine aminotransferase (IU/L)	53 (13–458)	81 (15–783)	< 0.01
Gamma-glutamyl transpeptidase (IU/L)	72 (11–786)	71 (16–990)	0.672
Hemoglobin (g/dL)	14.5 (6.5–17.5)	14.8 (9.2–18.7)	0.192
Platelet count (× 104/μL)	22.0 (4.0–47.1)	20.8 (5.0–37.7)	0.236
Triglyceride (mg/dL)	130 (31–1088)	145 (52–570)	0.021
Total cholesterol (mg/dL)	196 (101–290)	205 (103–370)	0.350
High-density lipoprotein cholesterol (mg/dL)	47 (22–85)	44 (14–86)	0.227
Low-density lipoprotein cholesterol (mg/dL)	116 (27–218)	124 (31–243)	0.073
Fasting blood sugar (mg/dL)	100 (70–237)	105 (65–287)	< 0.01
Glycated hemoglobin (%)	5.8 (4.4–10.8)	6.0 (4.3–12.6)	0.027
Ferritin (ng/mL)	191 (10–1472)	249 (1–2067)	< 0.01
NAFLD fibrosis score	− 2.415 (− 7.060 to 3.095)	− 1.550 (− 5.481 to 3.394)	< 0.01
Genetic variation			
Cases tested, (n)	110	204	
*PNPLA3* rs738409, GG (%)	41.8	41.7	1.000
*TM6SF2* rs58542926, non CC (%)	26.4	23.5	0.585
*HSD17B13* rs6834314, non AA (%)	56.4	45.7	0.078
Histopathological findings			
Cases examined, (n)	170	276	
Steatosis 3 (%)^c^	18.9	29.2	0.018
Ballooning 2 (%)^d^	22.5	29.9	0.099
Lobular inflammation 2–3 (%)^e^	23.7	44.2	< 0.01
NAFLD activity score 5–8 (%)	34.9	58.0	< 0.01
Fibrosis stage 3–4 (%)	22.4	38.4	< 0.01

Data are number of patients or median (range) values

*P* value by Mann–Whitney U test for continuous parameters and Fisher’s exact test for categorical parameters

^a^Lean NAFLD, BMI < 25.0 kg/m^2^, ^b^non-lean NAFLD, BMI ≥ 25.0 kg/m^2^, ^c^Steatosis 3, steatosis of ≥ 66% of hepatocytes, ^d^Ballooning 2, hepatocyte ballooning of many cells, ^e^Lobular inflammation 2–3, ≥ 2 foci per 200 × field

### Incidence of various events stratified by BMI

Table 3 lists the incidence of liver events, cardiovascular events, type 2 diabetes mellitus, and non-liver malignancies in patients with NAFLD. We analyzed the person-years method for patients with new onset during follow-up who did not have each disease before or at liver biopsy. In the lean NAFLD group, 6/155 (3.9%) patients developed liver cancer (rate per 1000 person years, 4.49). Furthermore, 4/148 (2.7%) patients confirmed to have no previous or current liver-related events at NAFLD diagnosis developed liver-related events (rate per 1000 person years, 3.08). Furthermore, 15/168 (8.9%) patients developed cardiovascular events, with a development rate per 1000 person years of 11.07. Further analysis showed 12/128 (9.4%) patients developed type 2 diabetes mellitus (rate per 1000 person years, 10.95) and 12/152 (7.9%) patients developed non-liver malignancies (rate per 1000 person years, 9.60). The liver cancer development rate per 1000 person years tended to be higher in the lean NAFLD group (4.49) than the non-lean group (1.76). On the other hand, the proportion of patients with T2DM was lower in the lean group than the non-lean group (10.95 *vs* 19.88).

**Table 3:** Incidence of liver events, cardiovascular events, type 2 diabetes mellitus, and non-liver malignancies in patients with NAFLD

Events	Overall		Lean NAFLD^a^		Non-lean NAFLD^b^	
	n/N (%)	1000 person years	n/N (%)	1000 person years	n/N (%)	1000 person years
Liver-related events	11/405 (2.7%)	3.72	4/148 (2.7%)	3.08	7/257 (2.7%)	4.23
Liver cancer	9/418 (2.2%)	2.96	6/155 (3.9%)	4.49	3/263 (1.1%)	1.76
Hepatic encephalopathy	6/444 (1.4%)	1.86	3/168 (1.8%)	2.11	3/276 (1.1%)	1.67
Esophagogastric varices	7/432 (1.6%)	2.22	3/163 (1.8%)	2.16	4/269 (1.1%)	2.26
Ascites	9/441 (2.0%)	2.80	4/167 (2.4%)	2.82	5/274 (1.8%)	2.78
Jaundice	3/442 (0.7%)	0.93	0/167 (0.0%)	0.00	3/275 (1.1%)	1.68
Cardiovascular events	36/443 (8.1%)	11.72	15/168 (8.9%)	11.07	21/275 (7.6%)	12.24
Type 2 diabetes mellitus	34/298 (11.4%)	15.43	12/128 (9.4%)	10.95	22/170 (12.9%)	19.88
Non-liver malignancies	26/406 (6.4%)	9.06	12/152 (7.9%)	9.60	14/254 (5.5%)	8.65

n; number of events, N; number of patients free or with the respective event at the time of NAFLD diagnosis

^a^Lean NAFLD, BMI < 25.0 kg/m^2^, ^b^non-lean NAFLD, BMI ≥ 25.0 kg/m^2^

### Mortality stratified by BMI

**Fig. 1:**
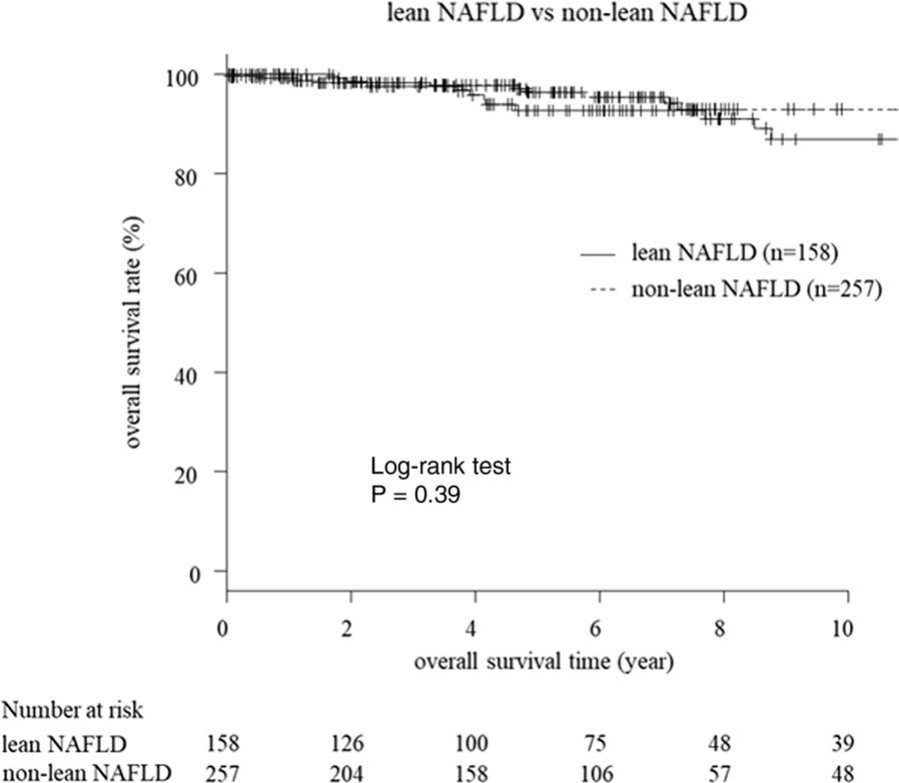
Kaplan–Meier curves for survival time in lean NAFLD and non-lean NAFLD. Kaplan–Meier analysis showed no significant difference in overall survival between the two groups (*P* = 0.39)

Table 4 lists the number and rate of mortality associated with liver-related events, non-liver cancer malignancies, and other events in patients with NAFLD. A person-year analysis was performed on patients who did not have the disease before or at the time of liver biopsy and died of the disease during follow-up. For the lean NAFLD group, 4/132 (3.0%) patients died during the study (per 1000 person-years, 3.32), with liver-related events in 3 (2.3%) (rate per 1000 person years, 2.49) and non-liver cancer malignancy in 1 (0.8%) (rate per 1000 person years, 0.83). Deaths from liver-related diseases tended to be more common in the lean NAFLD group than the non-lean NAFLD group, but the difference was not statistically significant. Kaplan–Meier analysis also showed no significant difference in overall survival between the two groups (*P* = 0.39) (Fig. 1).

**Table 4:** causes of mortality in patients with NAFLD

Cause of death	Overall		Lean NAFLD^a^		Non-lean NAFLD^b^	
	n/N (%)	1000 person years	n/N (%)	1000 person years	n/N (%)	1000 person years
Overall	9/360 (2.5%)	3.31	4/132 (3.0%)	3.32	5/228 (2.2%)	3.31
Liver-related events	5/360 (1.4%)	1.84	3/132 (2.3%)	2.49	2/228 (0.9%)	1.32
Non-liver malignancies	2/360 (0.6%)	0.74	1/132 (0.8%)	0.83	1/228 (0.4%)	0.66
Other events *	2/360 (0.6%)	0.74	0/132 (0.0%)	0.00	2/228 (0.9%)	1.32

n; number of events. N; number of patients with or without the respective event at the time of NAFLD diagnosis

^a^Lean NAFLD, BMI < 25.0 kg/m^2^, ^b^non-lean NAFLD, BMI ≥ 25.0 kg/m^2^

^*^Sepsis and interstitial pneumonia

### Non-invasive predictors of severe fibrosis (stage 3–4) in patients with lean NAFLD

Table 5 summarizes the baseline characteristics and laboratory data of the 170 patients with lean NAFLD (n = 132, with fibrosis stage 0–2, n = 38 for fibrosis stage 3–4). Age, history of liver cancer and prevalence of type 2 diabetes mellitus were significantly higher in patients with fibrosis stage 3–4. Furthermore, hemoglobin, platelet count, total cholesterol, and low-density lipoprotein cholesterol were significantly lower, while aspartate aminotransferase, fasting blood sugar, glycated hemoglobin, and NFS were significantly higher in patients with fibrosis stage 3–4. *PNPLA3* GG was significantly more frequent in the group of fibrosis stage 3–4. For histopathological findings, ballooning 2 and lobular inflammation 2–3 were significantly more frequent in the fibrosis score 3–4 group.

**Table 5:** Background factors in patients with lean NAFLD stratified by fibrosis stage

	Fibrosis stage 0–2 (n = 132)	Fibrosis stage 3–4 (n = 38)	*P* value
Age	49 (18–79)	67 (28–85)	< 0.01
Sex, male (%)	64.4	50.0	0.132
BMI (kg/m2)	23.1 (18.8–24.8)	23.3 (18.1–24.9)	0.388
History of liver cancer (%)	4.6	23.7	< 0.01
History of non-liver malignancy (%)	6.9	18.4	0.054
Type 2 diabetes mellitus (%)	16.2	50.0	< 0.01
Dyslipidemia (%)	25.8	36.8	0.220
Hypertension (%)	29.5	39.5	0.323
Hyperuricemia (%)	2.3	5.3	0.311
Smoking (%)	22.8	19.4	0.821
Albumin (g/dL)	4.1 (2.8–5.4)	4.1 (2.8–4.9)	0.083
Aspartate aminotransferase (IU/L)	36 (12–312)	46 (17–139)	< 0.01
Alanine aminotransferase (IU/L)	56 (13–458)	41 (17–280)	0.311
Gamma-glutamyl transpeptidase (IU/L)	79 (11–786)	63 (17–285)	0.184
Hemoglobin (g/dL)	14.7 (10.1–17.5)	13.9 (6.5–16.4)	< 0.01
Platelet count (× 10^4^/μL)	23.8 (9.8–47.1)	15.5 (4.0–32.8)	< 0.01
Triglyceride (mg/dL)	131 (31–1088)	125 (36–610)	0.287
Total cholesterol (mg/dL)	202 (101–290)	178 (101–280)	< 0.01
High-density lipoprotein cholesterol (mg/dL)	47 (23–85)	45 (22–85)	0.589
Low-density lipoprotein cholesterol (mg/dL)	117 (27–218)	95 (29–185)	< 0.01
Fasting blood sugar (mg/dL)	98 (70–159)	118 (76–237)	< 0.01
Glycated hemoglobin (%)	5.7 (4.7–9.3)	6.1 (4.4–10.8)	0.022
Ferritin (ng/mL)	180 (10–697)	231 (10–1472)	0.083
NAFLD fibrosis score	− 2.862 (− 7.060 to 1.566)	− 0.193 (− 4.723 to 3.095)	< 0.01
Genetic variation			
Cases tested, (n)	83	27	
*PNPLA3* rs738409, GG (%)	34.9	63.0	0.014
*TM6SF2* rs58542926, non CC (%)	25.3	29.6	0.802
*HSD17B13* rs6834314, non AA (%)	54.2	63.0	0.506
Histopathological findings			
Cases tested, (n)	132	37	
Steatosis 3 (%)	18.9	18.9	1
Ballooning 2 (%)	15.9	45.9	< 0.01
Lobular inflammation 2–3 (%)	18.9	40.5	< 0.01
NAFLD activity score 5–8 (%)	31.1	48.6	0.053

Data are number of patients or median (range) values

*P* value by Mann–Whitney U test for continuous parameters and Fisher’s exact test for categorical parameters

^a^Steatosis 3, steatosis of ≥ 66% of hepatocytes, ^b^Ballooning 2, hepatocyte ballooning of many cells, ^c^Lobular inflammation 2–3, ≥ 2 foci per 200 × field

All parameters that correlated strongly with others were considered confounding factors and excluded from statistical analysis. Thus, 14 non-invasive potential predictive factors of severe fibrosis (stage 3–4) were analyzed (Table 6). Univariate analysis identified four significant parameters; history of liver cancer, hemoglobin, NFS, and *PNPLA3*. These parameters were entered into multivariate analysis using the logistic regression analysis. The results identified NFS as a significant and independent factor determinant of development of severe fibrosis (stage 3–4) (≥ − 1.455, *P* < 0.01) (Table 6).

**Table 6:** Predictors of severe fibrosis (stage 3–4) in patients with lean NAFLD

	Univariate			Multivariate		
	OR	(95% CI)	*P* value	OR	(95% CI)	*P* value
Sex						
Female	1.8	(0.81–4.00)	0.132			
Male	1					
History of liver cancer						
Yes	6.37	(1.86–23.60)	0.001	2.05	(0.37–11.40)	0.411
No	1			1		
History on non-liver malignancy						
Yes	3.01	(0.88–9.93)	0.054			
No	1					
Dyslipidemia						
Yes	1.68	(0.72–3.83)	0.220			
No	1					
Hypertension						
Yes	1.55	(0.68–3.49)	0.323			
No	1					
Hyperuricemia						
Yes	2.37	(0.19–21.56)	0.311			
No	1					
Smoking						
No	1.22	(0.46–3.66)	0.821			
Yes	1					
Gamma-glutamyl transpeptidase (IU/L)						
< 71	1.35	(0.62–3.00)	0.464			
≥ 71	1					
Hemoglobin (g/dL)						
≤ 14.5	2.16	(0.97–5.03)	0.045	1.37	(0.43–4.36)	0.594
> 14.5	1			1		
Ferritin (ng/mL)						
≥ 180	1.16	(0.52–2.61)	0.711			
< 180						
NAFLD fibrosis score						
≥ − 1.455	16.49	(6.43–47.12)	< 0.01	14.60	(4.50–47.20)	< 0.01
< − 1.455	1			1		
*PNPLA3* rs738409						
GG	3.13	(1.18–8.74)	0.014	1.78	(0.58–5.44)	0.312
Non GG	1			1		
TM6SF2 rs58542926						
Non CC	1.24	(0.41–3.53)	0.802			
CC	1					
*HSD17B13* rs6834314						
Non AA	1.43	(0.54–3.94)	0.506			
AA	1					

Parameters that correlated significantly with other variables were considered confounding factors and excluded from statistical analysis

P values by logistic regression analysis

### Non-invasive predictors of survival in patients with lean NAFLD

**Fig. 2:**
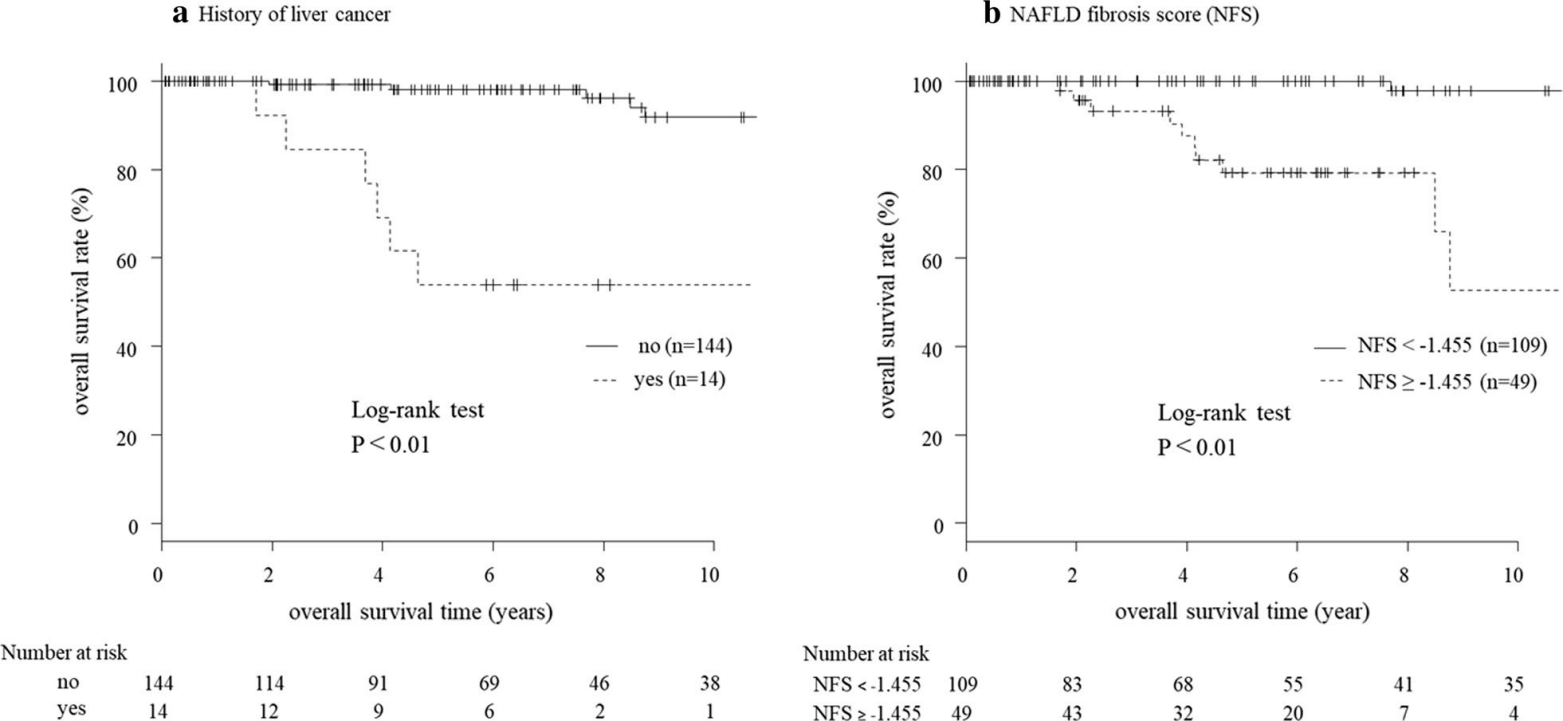
Kaplan–Meier curves for survival time in lean NAFLD stratified by **a** history of liver cancer and **b** NAFLD fibrosis score. The analysis showed a significantly shorter overall survival in patients with previous liver cancer (yes, *P* < 0.01) and with higher NFS (≥ − 1.455, *P* < 0.014)

Parameters that correlated strongly with others were considered confounding factors and excluded from statistical analysis. Thus, 14 non-invasive potential predictive factors of prognosis were analyzed (Table 7). Univariate analysis showed that history of previous liver cancer and NFS correlated significantly with survival. These two factors were entered into multivariate analysis using the Cox proportional hazards model. The analysis identified both parameters as significant and independent prognostic factors for lean NAFLD (yes for history of liver cancer, *P* < 0.01, NFS: ≥ − 1.455, *P* = 0.026) (Table 7). Furthermore, Kaplan–Meier analysis also showed that overall survival was significantly shorter in patients with previous liver cancer (*P* < 0.01) and in those with high NFS (≥ − 1.455, *P* < 0.014) (Fig. 2).

**Table 7:** Predictors of survival of patients with lean NAFLD

	Univariate			Multivariate		
	HR	(95% CI)	*P* value	HR	(95% CI)	*P* value
Sex						
Male	1.47	(0.45–4.83)	0.526			
Female	1					
History of liver cancer						
Yes	15.89	(4.62–54.62)	< 0.01	7.17	(1.76–29.23)	< 0.01
No	1					
History of non-liver malignancy						
Yes	2.89	(0.60–13.99)	0.186			
No	1					
Dyslipidemia						
No	3.88	(0.50–30.35)	0.196			
Yes	1					
Hypertension						
Yes	1.33	(0.39–4.53)	0.653			
No	1					
Hyperuricemia						
No	25,570,000	(0.00-∞)	0.998			
Yes	1					
Smoking						
No	1.09	(0.23–5.12)	0.916			
Yes	1					
Gamma-glutamyl transpeptidase (IU/L)						
≥ 71	1.18	(0.36–3.87)	0.786			
< 71	1					
Hemoglobin (g/dL)						
≤ 14.5	1.76	(0.51–6.01)	0.369			
> 14.5	1					
Ferritin (ng/mL)						
≥ 180	1.33	(0.40–4.40)	0.640			
< 180	1					
NAFLD fibrosis score						
≥ − 1.455	31.43	(3.90–253.2)	< 0.01	12.87	(1.35–122.30)	0.026
< − 1.455	1					
*PNPLA3* rs738409						
GG	2.55	(0.61–10.73)	0.202			
Non GG	1					
TM6SF2 rs58542926						
CC	2.19	(0.27–17.82)	0.463			
Non CC	1					
*HSD17B13* rs6834314						
Non AA	1.03	(0.252–4.066)	0.986			
AA	1					

Parameters that correlated significantly with other variables were considered confounding factors and excluded from statistical analysis

P values by Cox proportional hazards model

## Discussion

The clinical characteristics and prognostic factors of lean NAFLD remains unclear. In this retrospective study, we investigated the clinical features of histopathologically-confirmed cases of lean NAFLD and analyzed clinically useful parameters obtained non-invasively for the prediction of severe fibrosis and prognosis.

Our study included 170 patients with lean NAFLD and 276 with non-lean NAFLD. Lean NAFLD was associated with a lower complication rate of metabolic syndrome and better general biochemical data and pathological findings than the non-lean NAFLD. However, the rates of liver-related events, cardiovascular events, and malignancies were not different between the two groups, though the incidence of liver cancer was particularly higher in lean NAFLD. The overall survival rate was not significantly different between the two groups. Previous studies suggested that the metabolic profile of lean NAFLD is similar or slightly better than that of the non-lean NAFLD [19, 20], which was also noted in this study. The finding that patients with lean NAFLD are at higher risk of severe liver disease despite the lower prevalence of advanced fibrosis and NASH at baseline is paradoxical. Logically, this suggests that progression of liver fibrosis is faster in lean NAFLD than in NAFLD obese patients [9]. Further studies are needed to confirm this speculation.

Several studies have described age, diabetes, cirrhosis, low platelet count and low albumin levels as significant prognostic factors for NAFLD/NASH [21–28]. Although there are only a few reports on the prognostic factors of lean NAFLD among NAFLDs, one previous study identified fibrosis stage, hypertension, and age as independent prognostic factors [9]. As mentioned above, lean NAFLD patients have shorter history and less abnormal laboratory findings than those with non-lean NAFLD. Analysis of non-invasive predictors of fibrosis and prognosis of lean NAFLD is needed because early intervention is needed to improve prognosis. Such non-invasive markers should serve to: i) in primary care settings, identify the risk of NAFLD among individuals with increased metabolic risk; ii) in secondary and tertiary care settings, identify those with worse prognosis, e.g. severe NASH; iii) monitor disease progression; and iv) predict response to therapeutic interventions. Achieving these objectives could reduce the need for liver biopsy [8]. NFS or FIB-4 index are clinically useful tools for identifying NAFLD patients with higher likelihood of having bridging fibrosis (stage 3) or cirrhosis (stage 4) [29, 30]. The NAFLD fibrosis score (NFS) has the advantage that no special test items are included. We identified NFS of ≥ − 1.455 as a non-invasively measured parameter for the prediction of severe fibrosis in lean NAFLD. More importantly, for the first time, we found that NFS of ≥ − 1.455 is also a non-invasive independent and significant predictor of prognosis.

The present study has certain limitations. First, the median observation period was 4.6 years, which is a relatively short in prognostic studies. Second, the subject of this study were patient admitted to the Department of Hepatology of our hospital for liver biopsy. All subjects were Asians and admitted for the purpose of scrutiny of liver disease. Thus, a selection bias cannot be excluded. Further studies are needed that include patients of different races and healthy people followed for longer periods.

## Conclusions

Although patients with lean NAFLD had better histologic and biochemical profile compared to patients with non-lean NAFLD, it may be risky to end those follow-ups based on the lack of differences in prognosis between the two groups. In lean NAFLD, patients with NAFLD fibrosis score of ≥ − 1.455 or history of liver cancer should be followed-up carefully.

## Data Availability

The datasets generated and/or analyzed in the present study are available from the corresponding author on reasonable request.

## Abbreviations

BMI: Body mass index
CI: Confidence interval
HCV: Hepatitis C virus
HR: Hazard ratio
HSD17B13: Hydroxysteroid 17-beta dehydrogenase 13
NAFLD: Nonalcoholic fatty liver disease
NASH: Nonalcoholic steatohepatitis
NPR: Patient Register of Hospital Discharges
OR: Odds ratio
PNPLA3: Patatin-like phospholipase domain-containing 3
T2DM: Type 2 diabetes mellitus
TM6SF2: Transmembrane 6 superfamily member 2
